# Editorial: Tyrosine kinases and phosphatases in T- cell malignancies

**DOI:** 10.3389/fonc.2024.1372133

**Published:** 2024-02-02

**Authors:** Melania Tesio

**Affiliations:** U111, Lymphoma-Immuno Biology, Centre International de Recherche en Infectiologie, Lyon, France

**Keywords:** tyrosine kinase, tyrosine phosphatases, ALK (anaplastic lymphoma kinase), T-ALL, T-cell lymphomas

Tyrosine kinases (TKs) control a wide range of biological functions, including cell proliferation, differentiation and metabolism (1). Under normal physiologic conditions, TK activity is tightly balanced by tyrosine phosphatases (PTPs). This is crucial to guarantee the physiological development of thymocytes as well as the functions of peripheral T-cells. A most notable example in this context concerns the regulation of the TCR signaling. During T-cell development in the thymus, the TCR activation allows the selection of cells able to recognize non-self antigens and to tolerate self-antigens. In mature post-thymic cells, TCR engagement leds to T-cells activation, clonal expansion and differentiation into effector cells. In both developing and mature T-cells, TCR signaling is fined tuned by an interplay between phosphates and kinases, such as the phosphatase CD45 and the tyrosine kinase Lck, which initiates signal transduction downstream the TCR-CD3 complex. By de-phosphorylating Lck on distinct residues, CD45 positively regulates TCR activation in developing thymocytes (2, 3) while it negatively regulates TCR sensitivity in mature T-cells (4).

Genomic and nongenomic mechanisms eliciting the aberrant activation of tyrosine kinases and/or the loss of phosphatases activities perturb the physiological development of T-cells and their controlled proliferation, thus playing a significant role in the development of T-cell malignancies, a heterogeneous group of disorders resulting from the clonal evolution of dysfunctional T-cells at various stages of development. Inactivating mutations in CD45 are for instance being detected in T-cell acute lymphoblastic leukemia (T-ALL) (5), a T-cell malignancy arising from the transformation of immature thymic precursors. Within the malignancies arising from mature post-thymic cells, a notable example concerns the (2, 5) (p23;q35) translocation, which generates NPM-ALK, a chimeric tyrosine kinase driving the development of anaplastic large cell lymphoma (ALCL) (6).

Understanding the mechanisms which deregulate tyrosine kinases and phosphates signaling and their interplay is thus crucial to shed lights into the pathogenesis of T-cell malignancies and to develop novel targeted therapies. The collection of articles in this Research Topic highlights some of these aspects.

In their article, Mura et al. provide new insights into the oncogenic signaling elicited by NPM-ALK in anaplastic T-cell lymphoma. The authors demonstrated that, by activating STAT3, NPM-ALK represses CD45 expression, particularly of its CD45RO isoform. CD45 deregulation was consistent with the repression of the TCR signaling characterizing ALK-ALCL, and it was associated to an increased resistance to the ALK inhibitor crizotinib.

The deregulated ALK signaling in ALCL is also discussed by Piccaluga et al., who review altered tyrosine kinases signaling in most common forms of nodal peripheral T-cell lymphomas (PTCL). In addition to providing an overview of the signaling elicited by ALK fusion proteins, the authors report on the clinical activity of distinct ALK inhibitors. In a similar manner, after describing the alteration of JAK/STAT5 and PDGFR signaling in various PTCL forms, they authors comment on the effectiveness of small molecule inhibitors used in clinical settings to inhibit these pathways (i.e. ruxolinitib, dasatinib). Last but not least, the author report on the deregulation of tyrosine kinases involved in the TCR signaling, such as SYK and FYN, and on the therapeutic approaches undertaken to inhibit them.

In their study, Shang et al. report on a clinical case with relapsed/refractory ALK- anaplastic T-cell lymphoma. After achieving a 13-years disease free survival from an initial disease, the patient relapsed and manifested bone lesions as main clinical symptoms. The authors report a partial remission after two cycles treatment with a combination of brentuximab vedotin and a modified CHEP regimen (cyclophosphamide, adriamycin, etoposide, prednisolone) containing mitoxantrone hydrochloride liposome. After a maintenance therapy with oral chidamide, the authors report a disease-free survival of 16 months to date.

Whereas the first three articles of this Research Topic deal with mature T-cell malignancies, the last one focuses on T-cell acute lymphoblastic leukemia. The authors demonstrated that in this disease the E3 ubiquitin ligase TRIM37 promotes proliferation and apoptosis resistance as it ubiquitinates the dual lipid/tyrosine phosphatase PTEN (Qu et al.). In turn this lead to the down-regulation of this tumor suppressing protein, which plays important rollin T-ALL development (7).

In summary, this Research Topic bridges experimental data and clinical data which highlight de-regulated tyrosine kinase and phosphatase signaling to provide novel insights to foster the development of novel targeted therapies for T-cell malignancies.

## Author contributions

MT: Conceptualization, Writing – original draft, Writing – review & editing.
